# Association of fish oil containing lipid emulsions with retinopathy of prematurity: a retrospective observational study

**DOI:** 10.1186/s12887-022-03174-9

**Published:** 2022-03-02

**Authors:** Rongqiang Yang, Hao Ding, Jing Shan, Xiaole Li, Jian Zhang, Guanghui Liu, Hong Zheng, Yu Su, Hongyang Yao, Kemin Qi

**Affiliations:** 1 Department of Clinical Nutrition, Anhui Provincial Children’s Hospital, Wangjiang East Road 39, Hefei, 23000 Anhui China; 2 Beijing Paediatric Research Institute, Key Laboratory of Major Diseases in Children, Ministry of Education, Beijing Children’s Hospital, Capital Medical University, National Centre for Children’s Health, Beijing, 100045 China

**Keywords:** Retinopathy of prematurity, n-3 polyunsaturated fatty acids, Preterm infants, Fish oil, Lipid emulsions

## Abstract

**Background:**

Retinopathy of prematurity (ROP) remains a leading cause of childhood blindness worldwide. This study aimed to investigate whether supplementation of n-3 polyunsaturated fatty acids (n-3 PUFAs) in parenteral nutrition may have beneficial effects on ROP in preterm infants.

**Methods:**

A total of 89 preterm infants, admitted to Neonatal Intensive Care Unit (NICU) in Anhui Provincial Children’s Hospital from September 2017 to August 2020, were recruited in the study. Based on the medical documents, the subjects were categorised into two groups: administration of the fish oil emulsion (*n*=43) containing soy oil, medium-chain-triglycerides (MCT), olive oil and fish oil (6g/dL, 6g/dL, 5g/dL and 3g/dL respectively), and the soy oil emulsion (*n*=46) containing 10g/dL of soy oil and MCT each. At 4 weeks of hospitalization, ROP was screened and diagnosed. Fatty acids in erythrocytes were determined using gas chromatography.

**Results:**

The averaged birth weight and gestational age were 1594±296 g and 31.9±2.3 wk, 1596±263 g and 31.6±2.3 wk respectively for preterm infants in the fish oil group and soy oil group. After 4 to 6 weeks of hospitalization, among all the preterm infants, 52 developed ROP (all stages) indicating an incidence of ROP at 58.43%. Although the incidence of ROP with any stages showed no differences between the two groups, the severe ROP incidence in the group with fish oil emulsions (2.33%) was significantly lower than that in the group with soy oil emulsions (23.91%) (*P*<0.05). After 14 days of nutrition support, the preterm infants administered fish oil emulsions had an increase in erythrocyte DHA content, with a reduction in ratio of arachidonic acid (AA) to DHA and an increase of n-3 index.

**Conclusion:**

Supplementation of n-3 PUFAs through parenteral fish oil containing lipid emulsions resulted in an increase in erythrocyte DHA, and this might have beneficial effects on prevention of severe ROP in preterm infants.

## Background

Retinopathy of prematurity (ROP) is a potentially sight-threatening neurovascular disease which affects the retina in infants, especially in preterm infants [1–3]. During the past two decades, the accomplishment of neonatal health care system supports a higher survival rate of preterm infants than ever before, leading to an increased incidence of ROP worldwide. Although there has been considerable improvement on ROP with respect to etiology, pathogenesis and treatment, it remains a leading cause of childhood blindness worldwide [4]. Prematurity and long-term high oxygen supplementation have been demonstrated to be the major risk factors; whereas, maternal diseases (hypertension, diabetes mellitus and infection), caesarean section, premature rupture of membranes, respiratory distress syndrome (RDS), low Apgar score, patent ductus arteriosus (PDA), necrotising enterocolitis (NEC), intraventricular haemorrhage (IVH) and sepsis are considered as alternative risk factors [4, 5].

It is clear that docosahexaenoic acid (DHA) (20:6n-3), belonging to the series of n-3 polyunsaturated fatty acids (n-3 PUFAs), is an important component of retinal cell membrane and is required for photoreceptor cell development. The highest body concentration of DHA per unit area is found in the retinal disc membranes with a percentage of 30% of total retinal fatty acids [6–8]. Compared with full-term infants, preterm infants have lower plasma concentration and less body DHA storage, because they have missed important intrauterine accretion of nutrients including DHA in the third trimester. Furthermore, several investigations have found that preterm infants have capacity to synthesize DHA from precursor α-linolenic acid (ALA) (18:3n-3), but this endogenous synthesis may not be sufficient to cover the needs of the infant [9–11]. Consistent evidence demonstrates that n-3 PUFAs may act in a protective role against ischemia-, light-, oxygen-, inflammatory-, and age-associated pathogenesis of vasoproliferative and neurodegenerative retinal diseases such as ROP [8, 12–14].

Lipids are important components of parenteral nutrition to meet the requirements for essential fatty acids and high energy in preterm infants, who have limited endogenous lipid storage [15, 16]. Lipid emulsions are available for use as part of parenteral nutrition, including conventional lipid emulsions consisting of pure soybean oil, mixed lipid emulsions consisting of soybean oil plus medium-chain triglycerides (MCT) and/or olive oil, and most recently, a multicomponent lipid emulsion comprising 30% soybean oil, 30% MCT, 25% olive oil, and 15% fish oil [16]. Soybean oil-based lipid emulsions containing primarily n-6 PUFAs, have been demonstrated to contribute to increased body levels of proinflammatory cytokines and oxidative stress [16]. Multicomponent lipid emulsions comprising fish oil n-3 PUFAs, growingly accepted during the past decades, may be beneficial for paediatric patients by supplying a balanced ratio of n-6 to n-3 PUFAs [16–18], but more evidence is needed particularly for preterm infants, regarding changes in blood fatty acid levels and potential health risk without strong evidence of benefit [19–21]. Therefore, in this retrospective observational study, we investigated the effects of fish oil n-3 PUFA containing lipid emulsions on body accumulation of DHA, and their associations with ROP in preterm infants.

## Methods

### Subjects

Based on the estimated sample size (from 19 to 192) with α=0.05 and β=0.1, and occurrence rate of ROP in preterm infants, out of a total of 212 preterm infants admitted to Neonatal Intensive Care Unit (NICU) in Anhui Provincial Children’s Hospital from September 2017 to August 2020, 89 preterm infants were enrolled in the study. Inclusion criteria were as follows: BW < 2000 g and GA < 34 weeks, and supported with parenteral nutrition longer than 14 days. Exclusion criteria were as follows: being with one or more major congenital malformation, inborn metabolic disorders, and having signs or symptoms of congenital infection. While in hospital, the detailed information was recorded on the preterm infants’ gestational age (GA), delivery mode, premature rupture of membranes, birth weight (BW), gender, Apgar score, duration of oxygen use, biochemical parameters (glucose, creatinine, alanine aminotransferase, bilirubin, albumin), accompanied diseases (PDA, RDS, NEC, IVH, sepsis), and maternal diseases (hypertension, diabetes mellitus). Heparin-anticoagulant intravenous blood samples were drawn on admission within 24 hours (h) postnatally, as well as at two weeks after treatment. The later blood specimens were obtained 4 hours after the lipid infusion was completed. After centrifugation, the plasma separated was conducted to examine the biochemical parameters for clinical diagnosis. The blood cells were then stored at -80°C for associated studies. In the current study, the leftover blood cells were collected for analyzing fatty acids in red blood cells. The institutional Review Board/Ethics Committee of Anhui Provincial Children’s Hospital reviewed and approved this retrospective, observational study, and the legal guardians gave written consent for their participation.

Based on the medical documents, the subjects were categorised into two groups: fish oil emulsion group (*n*=43) in which infants was given parenteral Multi-oil emulsions, containing soy oil, MCT, olive oil and fish oil at concentrations of 6g/dL, 6g/dL, 5g/dL and 3g/dL respectively (Fresenius Kabi Austria GmbH); and soy oil emulsion group (*n*=46) in which infants was received intravenous long-chain-triglycerides (LCT)-MCT emulsions, containing 10g/dL of soy oil and MCT each (Fresenius Kabi China GmbH). The initial emulsion dose was 0.5 to 1.0 g of lipids per kg of body weight, and was increased by 0.5 to 1.0 g of lipids per kg body weight every 24 hours with a maximum of 3.0 to 3.5 g of lipids per kg of body weight/d. Enteral nutrition used either maternal or donor breastmilk and minimal enteral feeding was started within 3 h of birth and administered every 2 to 3 h (1 to 2 mL/meal) with a gradual increase in volume.

### Ophthalmologic Assessment

For all the preterm infants in hospital included, ROP was screened and diagnosed in terms of the classification for ROP with three Zones, five Stages (abnormal vascular response at the junction of the vascularized and avascular retina of retinopathy), and the presence or absence of Plus disease, according to the International Classification of Retinopathy of Prematurity. Severe ROP was defined as the level of Stage 3 or higher and plus disease, with a higher risk of an unwanted outcome [22, 23]. Based on the retinal proceeding from incomplete vascularization (no Stage) to descriptions of the retinal appearance at the junction between the vascularized and avascular retina, the Stages of ROP are classified as Stage 1 with a faint line, Stage 2 with a 3-dimensional ridge, Stage 3 with “extraretinal neovascularization” (i.e., intravitreal vasoproliferation) that grows from the ridge into the vitreous, Stage 4 with partial retinal detachmen, and Stage 5 with total retinal detachment [22, 23]. The screening examination, conducted by one ophthalmologist trained in the diagnosis of ROP, started at 4 weeks of postnatal age, and the follow-up examination was conducted at 6 weeks of postnatal age.

### Fatty acid analysis in erythrocytes

Fatty acids in erythrocytes were determined using gas chromatography. Fatty acid methyl esters from red blood cells were prepared according to a modified method of Lepage [24, 25]. The same procedure was followed as our previous described [26] on an Agilent 6890N GC system, and the quantity of fatty acids was expressed as the percent (%) (wt/wt) of the total fatty acids.

### Statistical Analysis

Continuous data were expressed as means±SD, and categorical data were expressed as percent (%). The t-test, Chi-square test, Mann-Whitney U-test or Fisher’s exact test was used as appropriate. Statistical analysis was performed on an Apple MacBook laptop by using Statistical Product and Service Solutions (SPSS) software, version 26. *P* <0.05 was considered as significant difference.

## Results

### General information for the preterm infants

The characteristic of subjects in this study was presented in Table 1. The preterm infants administered fish oil emulsions had a lower rate of caesarean section, compared to that with soy oil emulsions (*P*<0.05). No differences were found between the two groups of infants with regard to other parameters including BW, GA, gender, premature rupture of membranes, Apgar score, duration of oxygen use, biochemical parameters, accompanied diseases (PDA, RDS, NEC, IVH, sepsis), and maternal diseases (hypertension, diabetes mellitus).

**Table 1:** Clinical characteristics of preterm infants with soy oil emulsions and fish oil emulsions

Variables	Group with soy oil emulsions (*n*=46)	Group with fish oil emulsions (*n*=43)	*P* value
General information			
Birth weight (g)	1594±296	1596±263	0.674
Gestational age (wk)	31.9±2.3	31.6±2.3	0.543
Male gender, n (%)	26 (56.5)	27 (62.8)	0.547
Premature of rupture membranes (h)	27.91 (0-384)	41.88 (0-1440)	0.395
Caesarean section, n (%)	28 (60.9)	17 (39.5)	0.044
Accompanied diseases			
PDA, n (%)	4 (8.7)	7 (16.3)	0.277
NEC, n (%)	6 (13)	1 (2.3)	0.112
IVH, n (%)	29 (63)	27 (62.8)	0.980
Sepsis, n (%)	1 (2.2)	1 (2.3)	1.000
RDS, n (%)	26 (56.5)	30 (69.8)	0.197
Plasma biochemical parameters^a^			
Glucose (mmol/L)	3.05±1.89	2.27±1.58	0.570
Creatinine (μmol/L)	56.51±25.49	53.83±16.79	0.967
Alanine aminotransferase (μmol/L)	10.70±28.25	4.97±4.30	0.153
Bilirubin (μmol/L)	50.64±28.83	46.92±13.92	0.965
Albumin (g/L)	33.12±3.49	33.74±2.75	0.371
Maternal diseases			
Diabetes mellitus, n (%)	1 (2.2)	2 (4.7)	0.608
Hypertension, n (%)	10 (21.7)	7 (16.3)	0.513

^a^Examined within 24 h of admission in the first 48 h after birth and before parenteral nutrition started. *PDA* Patent ductus arteriosus, *NEC* Necrotising enterocolitis, *IVH* Intraventricular haemorrhage, *RDS* Respiratory distress syndrome

### ROP incidences in the preterm infants

After 4 weeks of hospitalization, among all the preterm infants, 52 developed ROP with any stages indicating an incidence of ROP at 58.43%. As shown in Table 2, the incidence of ROP showed no differences between the two groups. However, the incidence of severe ROP in the fish oil emulsion group (2.33%) was significantly lower than that in the soy oil emulsion group (23.91%) both at 4 weeks and 6 weeks after admission (*P*<0.05).

**Table 2:** Differences in ROP incidence in preterm infants between soy oil emulsion and fish oil emulsion administration

Variables	Group with soy oil emulsions (*n*=46)		Group with fish oil emulsions (*n*=43)	
	4 wk	6 wk	4 wk	6wk
ROP (any stages), n (%)	27 (58.70)	28 (60.87)	24 (55.81)	24 (55.81)
stage I	8	9	11	11
stage II	8	8	12	12
Severe ROP, n (%)	11 (23.91)	11 (23.91)	1 (2.33)*	1 (2.33)*

^*^Compared to the soy oil emulsions at the same time point, *P*<0.05

### Changes in erythrocyte fatty acids in the preterm infants

The fatty acid compositions in erythrocytes from the two groups of preterm infants with soy oil and fish oil emulsions were shown in Table 3. Before parenteral nutrition performed, no differences in each fatty acid composition were found between the two groups. However, after 14 days of nutrition support, the preterm infants administered fish oil emulsions had increases in contents of total n-3 PUFAs, DHA and n-3 index in erythrocytes, with a reduced ratio of arachidonic acid (AA) (20:4n-6) to DHA (*P*<0.05). Whereas, those given soy oil-based emulsions had increases in contents of total n-6 PUFAs, linoleic acid (LA) (18:2n-6), and ratio of n-6/n-3 PUFAs, concomitant with a reduction of total saturated fatty acids, palmitic acid (C16:0) and stearic acid (C18:0) (*P*<0.05).

**Table 3:** Comparison of erythrocyte fatty acids before and after administration of lipid emulsions in preterm infants

Variables	Before PN		After 14 d of PN			
	Group with soy oil emulsions (*n*=46)	Group with fish oil emulsions (*n*=43)	Group with soy oil emulsions *(n*=46)	*P* value*	Group with fish oil emulsions (*n*=43)	*P* value*
Total SFAs	55.98±5.87	55.10±6.32	50.98±2.82	0.000	53.30±6.05	0.257
C16:0	35.88±3.87	35.44±4.24	32.98±1.94	0.001	34.15±3.71	0.211
C18:0	20.10±2.25	19.66±2.38	18.01±1.28	0.000	19.15±2.49	0.411
Total MUFAs	16.92±2.50	17.20±2.04	18.14±2.44	0.052	18.31±2.38	0.032
C16:1n-9	1.03±0.44	1.25±0.43	0.97±0.43	0.589	0.95±0.37	0.004
C18:1n-9	15.88±2.66	15.95±2.14	17.17±2.42	0.049	17.36±2.30	0.049
Total n-6 PUFAs	20.10±5.38	21.16±5.24	24.45±2.77	0.001	21.23±5.01	0.830
C18:2n-6	5.01±2.74	5.85±2.91	9.70±2.74	0.000	7.40±3.36	0.102
C18:3n-6	0.21±0.06	0.20±0.05	0.19±0.05	0.850	0.19±0.06	0.478
C20:4n-6	14.49±4.73	14.60±4.79	14.35±2.97	0.827	13.23±4.30	0.135
C22:5n-6	0.39±0.20	0.51±0.18	0.21±0.17	0.221	0.41±0.20	0.395
Total n-3 PUFAs	7.05±1.28	6.58±1.32	6.49±1.45	0.073	7.18±1.87	0.026
C18:3n-3	2.80±0.38	2.55±0.42	2.51±0.79	0.206	2.38±0.80	0.285
C20:5n-3	2.42±0.62	2.33±0.71	2.47±0.65	0.599	2.42±0.77	0.460
C22:6n-3	1.83±0.73	1.71±0.48	1.52±0.64	0.103	2.37±0.55	0.031
n-6/n-3 PUFAs	2.85±0.69	3.21±0.59	3.77±1.24	0.004	2.96±1.73	0.093
AA/DHA	7.93±4.71	8.54±2.38	9.46±2.53	0.137	5.57±2.18	0.047
n-3 index	4.25±1.08	4.04±1.21	3.98±1.16	0.062	4.79±1.09	0.037

*PN* Parenteral nutrition. ^*^Compared to the same lipid emulsions before PN administration

## Discussion

It has been increasingly recognized that ROP differs worldwide and tailored screening and treatment approaches are needed to reduce aberrant vasoproliferation and facilitate physiologic retinal vascular development in infants [4]. N-3 PUFAs are essential for normal retinal development and appear to play a protective role against retinal neovascularization and visual damage, if provided with n-3 PUFA-enriched formulas in preterm neonates [14]. DHA and its substrate eicosapentaenoic acid (EPA) (20:5n-3), being regulators for transcription factor, and parent fatty acids for neuroprotectin D1 and a family of eicosanoids, have beneficial effects on ischemia, oxidative stress, inflammation and cellular signaling mechanisms, influencing retinal cell gene expression and cellular differentiation, as well as activating molecules implicated in the pathogenesis of vasoproliferative and neurodegenerative retinal diseases such as ROP [14]. In our study, we found that intravenous fish oil containing lipid emulsions increased erythrocyte DHA content with no changes in EPA content and reduced the incidence of severe ROP in preterm infants, compared to the traditional soy oil lipid emulsions. Meanwhile, the beneficial effects of administration of lipid emulsions containing fish oil on retina has also been associated with the higher vitamin E/α-tocopherol levels compared with other lipid emulsions [27].

Concerning the effects of n-3 PUFA supplementation on ROP in preterm infants, no final conclusions have yet been reached. In a prospective cohort study, it is found that a time-dependent accumulation of AA at the expense of DHA seems to occur in utero in erythrocytes of preterm infants who will develop ROP, thus reinforcing the beneficial properties of DHA on this disease [28]. In other observational or prospective randomized studies, the use of intravenous fish oil-containing lipid emulsions is associated with a lower incidence of ROP or severe ROP and decreased need for laser or bevacizumab treatment in preterm infants [29–31], which is in keeping with our findings. Inconsistently, preterm infants treated with fish oil containing emulsions did not differ for any or severe ROP, as well as for bronchopulmonary dysplasia, NEC, PDA, or sepsis, and the incidence of cholestasis in extremely premature infants [32, 33]. Therefore, evidence is insufficient to determine with any certainty if fish oil emulsions offer advantage in prevention or resolution of ROP or in any other clinical outcome, and a large multicenter randomized clinical trial is required [18, 34]. Inconsistencies between studies may be ascribed to baseline differences in body n-3 PUFAs, different dosages used and duration, other nutrient deficiencies and lack of investigating interaction effects of gender and age [35]. In addition, our study presented that cesarean section is significantly higher in the soy oil group than that in the fish oil group. Recently, it is reported that babies delivered by caesarean section had a reduced risk of developing ROP compared with those delivered vaginally [16]. Thus, it was sensible for the lower incidence of severe ROP in the fish oil group with a lower cesarean section.

The accretion of PUFAs including n-3 series as well as n-6 series in the fetus increases exponentially from the 30th to the 38th week during pregnancy, and continues during the first 3 years after birth, and thus preterm infants may be in more disadvantage compared to term infants if n-3 PUFAs provided insufficiently after birth [36]. It has been reported that preterm infants fed milk with a DHA content 2-3 times higher than the current concentration in infant formulas have better neurologic outcomes in early life, and thus suggesting that human milk and preterm formula should contain approximately 1.5% of fatty acid as DHA to compensate for the early DHA deficiency [37, 38]. Herein, our results showed that the erythrocyte DHA content was increased with parenteral administration of the fish oil containing emulsions to preterm infants, instead of the soy oil lipid emulsions, which caused an increase in LA and total n-6 PUFA content in erythrocytes. Similarly, providing a target dose of 3 to 3.5 g/kg/day of lipid emulsions containing 15% of fish oil beneficially modulates the DHA profile, but providing lipid emulsions containing 10% fish oil at a dose of ≤2 g/kg/day fails to increase circulating DHA [39]. To note, intake or supplementation of either the quantity of n-3 PUFAs or the ratio of n-6/n-3 PUFAs determines the concentration of DHA in erythrocytes and tissues, and its health effects. In the current study, the ratio of n-6/n-3 PUFAs for the soy oil emulsions and fish oil emulsions was 8:1 and 4:1 respectively. In term infant formula, ratios of n-6 to n-3 PUFAs around 7:1 have been most commonly used, and preterm formulas usually have the ratios ranging between 5:1 and 15:1 [40]. Several expert groups recommend that infants receive at least 0.3% DHA and at least 0.3% AA, with a ratio of AA to DHA from 1:1 to 2:1 in infant feedings, is associated with improved visual and cognitive outcomes [41].

In addition to DHA, EPA is found to be beneficial for body health by antagonizing arachidonic acid (AA) derived eicosanoids, which is associated with retinal neurovascular impairment [8]. The n-3 index, the percentage of EPA plus DHA in erythrocytes, represents a human body’s status in EPA and DHA. The compiling recent data supports the target range for the omega-3 index of 8–11% in adults and pregnant mothers [42]. In children and adolescents, daily supplementation of ≥450 mg DHA + EPA and an increase in the n-3 index to >6% makes it more likely to show efficacy on cognitive development [43]. In the current study, the n-3 index was elevated to 4.79% by the use of fish oil containing lipid emulsions with no changes in erythrocyte EPA content, but it was still less than the idea value (6%) for children [43]. This might be resulted from the daily supplementation of less DHA+EPA (<157 mg/kg/day) in the lipid emulsions used. In consistency, other reports demonstrated that extremely premature infants on the same fish oil containing lipid emulsions with ours had significantly elevated fraction of EPA and slightly increased DHA fraction with the n-3 index of 4.03% at 14 days [27]. Next, supplementation of higher quantities of DHA+EPA using new lipid emulsion formulas and the optimal n-3 index need to be focused to elucidate their effects on the growth and development of infants including preterm babies.

To be noteworthy, evaluating postnatal AA status after birth and correcting its deficiency in addition to EPA and DHA need to be strengthened for ROP prediction and prevention, because AA is indispensable in the vasculature and in specific aspects of immunity, being functioned as a precursor for leukotrienes, prostaglandins, and thromboxanes, collectively known as eicosanoids in infant development [44]. The low postnatal AA levels has been proved to be strongly associated with the development of ROP (any stage of ROP and severe ROP) [45]. Furthermore, a randomized clinical trial study found that, compared with standard of care, enteral simultaneous supplementation of AA and DHA lowered the risk of severe ROP by 50% and showed overall higher serum levels of both AA and DHA [46]. In this study, erythrocyte AA content was not altered by parenteral administration of either soy oil or fish oil lipid emulsions, because body AA content is usually stable owing to the higher ability for AA synthesis from its precursor LA [40]. Nonetheless, the best clinical approach to PUFA supplementation and n-6 to n-3 PUFA (or AA to DHA) ratio are still far from evident, and requires in-depth investigation on specific fatty acid supplementation in the context of other fatty acids [40].

Administration of fish oil emulsions to preterm infants reduced the incidence of severe ROP with increased erythrocyte DHA concentration. Unfortunately, the current study was a retrospective observational study with a smaller sample size. To provide a more conclusive picture, future trials should employ larger sample size in prospective randomized studies with long-term follow-up and should focus on supplementation of higher quantities of DHA+EPA. Meanwhile, the contribution of enteral feeding to fatty acid concentrations in erythrocyte was not investigated. Finally, the averaged gestational age for preterm infants was 31 weeks, meaning more mature babies included in this study. Thus, study on preterm infants with gestational age less than 28 weeks should be highlighted as a future research perspective.

## Conclusion

In summary, administration of fish oil containing lipid emulsions increased erythrocyte DHA content with a reduction in AA/DHA ratio and an increase of n-3 index, even though EPA and AA content in erythrocytes was not changed, and consequently had beneficial effects on incidence of severe ROP in preterm infants.

## Data Availability

The datasets used and/or analyzed during the current study are available from the corresponding author on reasonable request.
